# Across the multiverse: exploring a diverse set of specifications related to cross-sectional and prospective associations between adolescent alcohol use and emotional problems

**DOI:** 10.1017/S0033291724002502

**Published:** 2024-12

**Authors:** J. Halladay, R. Visontay, T. Slade, E. K. Devine, S. Smout, J. L. Andrews, K. E. Champion, M. Teesson, M. Sunderland

**Affiliations:** 1 School of Nursing, McMaster University, Ontario, Canada; 2 The Matilda Centre for Research in Mental Health and Substance Use, University of Sydney, New South Wales, Australia; 3 Peter Boris Centre for Addictions Research, McMaster University/St. Joseph's Healthcare Hamilton, Ontario, Canada; 4 Department of Experimental Psychology, University of Oxford, Oxford, UK; 5 School of Public Health, University of Sydney, New South Wales, Australia

**Keywords:** adolescent, alcohol drinking, anxiety, depression, multiverse, psychological distress

## Abstract

**Background:**

The relationship between adolescent alcohol use and emotional problems remains unclear and contradictory. These inconsistencies may in part be due to differences in the measurement and operationalization of alcohol use and emotional problems across studies, as well as confounder selection and missing data decisions. This study explores the associations between common specifications of adolescent alcohol use and emotional problems in a large sample of adolescents.

**Methods:**

A multiverse analysis (also known as specification curve analysis or vibration of effects) was done with 7680 unique model specifications in a large longitudinal sample of 6639 Australian adolescents (aged ~14.7–15.7, 2021–2022).

**Results:**

While alcohol use and emotional problems nearly universally co-occurred in minimally adjusted cross-sectional models (98–99%), the operationalization of emotional problems, temporality of prospective relationships, and choice of confounders substantially impacted findings. Emotional problems appeared to predict later alcohol use more-so than the reverse, depression-focused measures yielded more consistent associations with alcohol use than anxiety-focused measures, and certain confounders (i.e. conduct, ADHD, smoking) explained most of the associations between adolescent alcohol use and emotional problems. Missing data decisions and whether outcomes were modelled continuously *v.* dichotomously had minimal impact on findings.

**Conclusions:**

While adolescent alcohol use and emotional problems commonly co-occur, inconsistencies in the magnitude, direction, and significance of effects are closely tied to researcher decisions that are often made arbitrarily.

## Background

Adolescence is a sensitive period during which most substance use (particularly alcohol use) and emotional problems (such as depression, anxiety, distress, and general internalizing symptoms) emerge (Solmi et al., 2021). These issues often co-occur in general population and clinical adolescent samples (Halladay, MacKillop, Munn, Amlung, & Georgiades, 2022; Hawke, Koyama, & Henderson, 2018; Suntharalingam et al., 2022), though the specific nature of the association between alcohol use and emotional problems remains unclear and contradictory. These inconsistencies may, in part, be due to differing researcher decisions regarding the conceptualization, measurement, and directionality of associations between alcohol use and emotional problems across studies. Contemporary statistical frameworks have been built to account for and uncover the impact of these researcher choices – which can be arbitrary – on the stability or robustness of effects across different specifications between alcohol use and emotional problems (Patel, Burford, & Ioannidis, 2015; Simonsohn, Simmons, & Nelson, 2020; Steegen, Tuerlinckx, Gelman, & Vanpaemel, 2016). Given the global importance of preventing alcohol use harms and emotional problems (United Nations Department of Economic and Social Affairs, 2015), a more robust understanding of their relationship is needed, considering factors like the timing and direction of associations, choice of confounders, and operationalization of alcohol use and emotional problems.

Large longitudinal datasets are powerful tools for exploring these relationships. However, the hypothesized causal relationship between alcohol use and emotional problems, which informs a study's chosen statistical model, may impact the detection and magnitude of associations. Three core hypotheses explain the co-occurrence of these problems. First, emotional problems may lead to alcohol use, often attributed to psychosocial pathways. Some studies suggest certain types of emotional problems predict early alcohol use and related problems later in life, though these associations are nuanced and inconsistent (Dyer, Easey, Heron, Hickman, & Munafò, 2019; Hussong, Ennett, Cox, & Haroon, 2017). Second, adolescent alcohol use may contribute to the development or worsening of emotional problems, often attributed to social, cognitive, or neurobiological pathways. Existing longitudinal studies reveal weak/negligible associations between adolescent alcohol quantity-frequency measures and later depression and anxiety (Cochrane Canada, 2022; McCabe, Brumback, Brown, & Meruelo, 2023). Third, alcohol use and emotional problems may share common risk and protective factors, leading to their co-occurrence due to confounding. Common confounders across studies include demographics, other substance use, and externalizing problems (Cochrane Canada, 2022; Dyer et al., 2019; Hussong et al., 2017; Ning, Gondek, Patalay, & Ploubidis, 2020). As such, researcher decisions pertaining to the selection of independent, dependent, and confounding variables may contribute to existing inconsistencies.

The operationalization of key constructs may also drive inconsistencies. Across existing studies, adolescent alcohol use is measured in diverse ways, from initial sipping to the diagnosis of alcohol use disorder (AUD). Common ways of operationalizing adolescent alcohol use include full standard drink consumption (prevalence and frequency), binge drinking (often defined as 5+ drinks/occasion), alcohol volume (frequency × quantity), and alcohol-related problem scales and diagnoses (Cochrane Canada, 2022; Dyer et al., 2019; Ning et al., 2020). Some researchers place emphasis on the age of initiation, particularly by age 14, as studies have shown earlier onset to strongly correlate with suicidality and longer-term alcohol problems (Ahuja, Awasthi, Records, & Lamichhane, 2021; Gardner, Stockings, Champion, Mather, & Newton, 2024; Lee et al., 2024). Additional inconsistencies may arise due to considerable variability in recall periods.

Existing research suggests that decisions about how to operationalize alcohol use may impact the nature of the associations found. For instance, a previous systematic review found a more consistent link between early initiation of alcohol use and depression compared to other aspects of alcohol use, such as alcohol problems, quantity-frequency, and AUD (Hussong et al., 2017). Conversely, another review found adolescent anxiety to be associated with later AUD, but found inconsistent evidence regarding anxiety's connection to other alcohol quantity-frequency variables (Dyer et al., 2019). A recent meta-analysis, predominantly focused on adults, revealed that individuals with mood or anxiety disorders had twice the odds of AUD compared to those without such disorders (Puddephatt, Irizar, Jones, Gage, & Goodwin, 2022). However, the direction and magnitude of effects was inconsistent for other quantity-frequency measures of alcohol use. This highlights the need for further investigation into the causes, correlates, and consequences of various facets of adolescent alcohol use in relation to emotional problems.

The term ‘emotional problems’, defined in the current study as encompassing depression, anxiety, psychological distress, and general internalizing-related factors, can be measured using various symptom scales and/or diagnostic assessments. While evidence suggests that these emotional problems may be better understood as a general internalizing factor (Watson et al., 2022), various sub-domains of emotional problems exhibit distinct associations with alcohol related factors (Dyer et al., 2019; Hussong et al., 2017; Ning et al., 2020). For instance, measures of depression predict later alcohol use and related problems more consistently than anxiety and general internalizing related measures (Greenwood et al., 2021; Hussong et al., 2017; Ning et al., 2020). The nature of these relationships may also depend on how emotional problems are assessed, such as differentiating between symptoms, a spectrum of severity, clinical thresholds, or meeting diagnostic criteria (Dyer et al., 2019; Hussong et al., 2017; Ning et al., 2020).

To date, no study has quantitatively evaluated the potential impact of different researcher decisions on the significance and magnitude of the association between adolescent alcohol use and emotional problems. In the present study we therefore explore the overall and specific associations between adolescent alcohol use and emotional problems in a large sample of adolescents across Australia. We do this by applying a contemporary framework for quantifying sensitivity to alternative specifications known as multiverse analysis (Steegen et al., 2016), with complementary approaches existing within specification curve (Simonsohn et al., 2020) and vibration of effects analyses (Patel et al., 2015). These allow us to examine and report all non-redundant, reasonable, and justifiable measurement and analytic specifications, and identify the consequences of these specification decisions. Ultimately, these three analytical approaches have a shared goal of summarizing effects across various sets of sensitivity analyses based on varying research or design decisions. In brief, the underlying motivation of multiverse analysis (Steegen et al., 2016) is to explore effects across the ‘multiverse’ of possible combinations to increase transparent reporting and identify key choices or aspects of relationships. Within this framework, each unique combination of specifications is considered to be an analytic ‘universe’ (which corresponds to a single regression model), and here, alcohol use–emotional problems analyses are explored for each universe before interpreting the overall set of results. For specification curve analysis (Simonsohn et al., 2020), the focus is on visualizing the range of all estimated effects in a ‘curve.’ For vibration of effects analysis (Patel et al., 2015), the main focus is on exploring all justifiable sets of confounders and how different confounder adjustments impact effect sizes. All of these approaches are applied in a single dataset, so we can analyze and directly compare a range of specifications (or models) within the same dataset. Specifically, this study identifies: (1) the overall association between alcohol and emotional problems among adolescents using common measurement and modelling specifications; (2) specifications that yield the strongest (and weakest) association(s); (3) the impact of frequently referenced confounding variables; and (4) the magnitude of difference between associations modelled cross-sectionally *v.* prospectively.

## Methods

### Data

This is a secondary analysis of data from the Health4Life Study, a cluster-randomized controlled trial of a school-based eHealth intervention targeting lifestyle risk behaviors. The Health4Life study recruited 6639 students aged 11–14 (average 12.7) in 71 schools across three Australian states (New South Wales, Queensland, and Western Australia) (Teesson et al., 2020). Baseline data were collected in 2019 (T1) with follow-ups at post-intervention (~7 weeks, T2), 12 (T3), 24 (T4), and 36 months (T5). Given the peak age of onset for mental disorders is 14.5 years of age (Solmi et al., 2021) and the average age of onset of alcohol use among young Australian's was 16.2 in 2019 (Australian Institute of Health and Welfare, 2020), this paper focused on data collected when students were on average 14.7–15.7 years of age to maximize the prevalence and variability in alcohol use and emotional problems. Prospective analyses were conducted with 24-month data (T4, mean age = 14.7, ~year 9) predicting 36-month outcomes (T5, mean age = 15.7, ~year 10) and cross-sectional analyses were based on 36-month data (T5). The overall response rate was 75.4 and 66.8% at 24-months and 36-months, respectively. Further, given the intervention did not demonstrate effects in modifying alcohol use or emotional problems by 24-months (Champion et al., 2023; Smout et al., 2024), students participating in both control and intervention arms were included, adjusting for trial arm.

### Parameters of interest and their specifications

#### Alcohol

Alcohol related specifications in this study included: past 6-months full standard drink (yes/no), past 6-months monthly or more drinking (yes/no), past 6-months frequency of drinking (never, <monthly, 1–2/month, 2–3/month, weekly, daily/almost daily), past 6-months binge drinking (yes/no), past 6-months monthly or more binge drinking (yes/no), past 6-months alcohol volume (frequency × quantity), alcohol-related harms as per a summative score on the Brief Rutgers Alcohol Problems Index (Earleywine, LaBrie, & Pedersen, 2008), and endorsing drinking a full standard drink in the past 6-months at any point in the study ⩽14 years of age (yes/no).

#### Emotional problems

Four domains of emotional problems were captured continuously and dichotomously by four commonly used scales. First, non-specific psychological distress was measured using the 6-item Kessler-6 (K6) that asks about frequency of feeling nervous, hopeless, restless or fidgety, depressed, that everything was an effort, and worthless over the past 4 weeks (Kessler et al., 2002). Item responses were summed ranging from 0–24 where higher scores reflect greater distress, with scores ⩾13 indicative of serious psychological distress. General internalizing problems were measured with the 5-item emotion symptoms subscale on the Strengths and Difficulties Questionnaire (SDQ-E; (Goodman, Meltzer, & Bailey, 1998). Scores were summed ranging from 0–10, with scores ⩾7 indicative of a problematic level of symptoms. Symptoms of depression were measured with an adapted 8-item version of Patient Health Questionnaire for adolescents (PHQ-A) that asks about the frequency of depressive symptoms over the past 7 days (Johnson, Harris, Spitzer, & Williams, 2002). The 9th item regarding suicidal ideation was dropped as per requests from ethics; notably, previous evaluations indicate comparable results and psychometric properties for the 8- and 9-item versions (Wu et al., 2020). Item responses were summed ranging from 0–24 where higher scores indicate more symptoms, with scores ⩾10 indicative of moderate to severe depressive symptoms. Symptoms of anxiety were measured with the 13-item PROMIS-Anxiety Pediatric Scale asking about frequency of symptoms over the past 7 days (Irwin et al., 2010). Item responses were summed ranging from 13–65 where higher scores indicate greater severity of anxiety, with scores ⩾34 indicative of moderate to severe anxiety symptoms.

#### Confounders

Five sets of confounders were created based on the most commonly included confounders in studies included in related systematic reviews (Cochrane Canada, 2022; Dyer et al., 2019; Hussong et al., 2017; Ning et al., 2020) and the foundational goals and hypotheses of the Health4Life trial (Teesson et al., 2020). Sets of confounders include: (1) demographics, (2) smoking, (3) conduct problems, (4) ADHD symptoms, and (5) other health behaviors including physical activity, screen time, sleep, and diet. Additionally, in prospective models, T4 emotional problems were controlled for when examining associations between T4 alcohol and T5 emotional problems (e.g. controlling for autocorrelation, or pre-existing levels). Similarly, T4 alcohol variables were controlled for when examining association between T4 emotional problems and T5 alcohol-related outcomes. In total, we evaluated 16 different confounder combinations (i.e. all combinations where demographics are always included, apart from the unadjusted model). See Table 1 for more details.

**Table 1.** Summary of multiverse specifications

**Alcohol Specifications**		
1	Past 6-months any alcohol use	*‘Have you had a full standard alcoholic drink* (*see alcohol chart*) *in the past 6 months?’* (0 = no, 1 = yes)
2	Past 6-months ~monthly alcohol use	*‘How often did you have a standard alcoholic drink of any kind in the past 6 months?’* (0 = never or less than monthly, 1 = once a month or more)
3	Past 6-months frequency of alcohol use	Standardized: ‘*How often did you have a standard alcoholic drink of any kind in the past 6 months?’* (0 = never, 1 = less than monthly, 2 = once a month, 3 = 2-3 times, 4 = weekly, 5 = daily or almost daily)
4	Past 6-months binge drinking	‘*How often did you have 5 or more standard alcoholic drinks on one occasion in the past 6 months?’* (0 = never, 1 = less than monthly or more)
5	Past 6-months ~monthly binge drinking	‘*How often did you have 5 or more standard alcoholic drinks on one occasion in the past 6 months?’* (0 = never or less than monthly, 1 = once a month or more)
6	Past 6-months average monthly alcohol volume (in grams/centilitres)	Natural log transformed and standardized: Frequency × quantity × 10 g of alcohol per standard drink Frequency: ‘How often did you have a *standard alcoholic drink* of any kind in the past 6 months?’ 0 = never, 0.5 = less than monthly, 1 = once a month, 2.5 = 2-3 times, 4 = weekly, 28 = daily or almost daily (note: frequency coded as the mid-point of the response option) Quantity: ‘In the past 6 months, how many *standard drinks* did you have on a typical day when you were drinking alcohol?’ 0 = none, 1.5 = 1-2, 3.5 = 3-4, 5.5 = 5-6, 8 = 7-9, 10 = 10 + note: frequency coded as the mid-point of the response option)
7	Past 6-months alcohol problems	Standardized: Brief Rutgers Alcohol Problems Index score
8	Early age of onset	Any alcohol use in the past 6 months reported ⩽14 years of age at any point in the study (0 = no, 1 = yes)
**Emotional Problems Specifications**		
1	Psychological distress symptoms	Standardized: K6 continuous score
2	High psychological distress	K6 ⩾13 (0 = No, 1 = Yes)
3	Emotional symptoms	Standardized: SDQ-E emotional symptoms continuous score
4	Abnormal emotional symptoms	SDQ-E emotional symptoms ⩾7 (0 = No, 1 = Yes)
5	Depressive symptoms	Standardized: PHQ-A continuous score
6	Moderate to severe depressive symptoms	PHQ-A ⩾10 (0 = No, 1 = Yes)
7	Anxiety symptoms	Standardized: PROMIS-A continuous score
8	Moderate to severe anxiety symptoms	PROMIS-A ⩾34 (0 = No, 1 = Yes)
**Confounder Specifications** Sets for analysis include: none (unadjusted), 1 (minimally adjusted), 1 + 2, 1 + 3, 1 + 4, 1 + 5, 1 + 2 + 3, 1 + 2 + 4, 1 + 2 + 5, 1 + 3 + 4, 1 + 3 + 5, 1 + 4 + 5, 1 + 2 + 3 + 4, 1 + 2 + 3 + 5, 1 + 3 + 4 + 5, 1 + 2 + 3 + 4 + 5		
1	Demographics	Sex, family affluence, cultural and linguistic diversity
2	Smoking	*‘In the past 6 months, have you tried cigarette smoking, even one or two puffs?’* (0 = No, 1 = Yes)
3	Conduct	SDQ conduct problems subscale continuous score
4	ADHD	SDQ hyperactivity/inattention subscale continuous score
5	Health Behaviors	Physical activity: Number of days engaging in 60 min or more of moderate to vigorous physical activity in the past 7 days (0-7). Sedentary screen time: Average time (hours) in the past 7 days spent sitting or lying down while using an electronic device during free time, excluding homework. Sleep: Whether adolescents were meeting sleep recommendations of 8-10 h per night based on mean sleep duration derived from a 6-item scale asking about bedtime, time attempted sleep, time taken to fall asleep, time awake, and time out of bed. Sugar-sweetened beverages: Usual consumption of soft drinks, cordials, or sports drinks per week operationalized as 0 = <1 cup per week, 1 = 2–4 cups per week, 2 = 5 or more cups per week.
**Missing data**		
1	Complete case	Listwise deletion
2	Multiply imputed	Averaged across 20 imputations
**Timing and implied direction of associations**		
1A	Cross-sectional	T5 alcohol variables predicting emotional problem variables
2A	Prospective	T4 alcohol variables predicting T5 emotional problems, adjusting for T4 emotional problems
1B	Cross-sectional	T5 emotional problems variables predicting alcohol variables
2B	Prospective	T4 emotional problems predicting T5 alcohol variables, adjusting for T4 alcohol variables

To note, each ‘universe’ includes 1 alcohol specification, 1 emotional problem specification, 1 set of confounder(s), and 1 missing data strategy.

#### Missing data

Similar to methods used in a prior multiverse analysis (Barendse et al., 2022), missing data were multiply imputed (*n* = 20) and then combined into a single dataset for inclusion in the multiverse analysis by taking the mean across imputations. Missing data was imputed using a multilevel fully conditional specification approach in BLIMP imputation software (Keller & Enders, 2021) with imputation models including all multiverse variables across all Health4Life timepoints. As such, a missing data specification was included to explore models with complete cases only *v.* multiple imputed data.

### Statistical analysis

We conducted a multiverse analysis (Steegen et al., 2016) supplemented with tools from similar statistical frameworks (i.e. specification curve analysis (Simonsohn et al., 2020) and vibration of effects (Patel et al., 2015)). Methods and code used for this paper come from previous multiverses (Barendse et al., 2022; Visontay et al., 2023) using R version 4.3.2 with packages *multiverse* (Sarma, 2023) and *specr* (Masur & Scharkow, 2023). Two multiverses were estimated based on different implied directions of associations: (A) alcohol variables predicting emotional problems, and (B) emotional problems predicting alcohol variables. See details in Table 1. All of the reasonable and possible ‘universes’ are modeled and then pooled for interpretation, which is called a ‘multiverse.’ The total multiverse of specifications for Multiverse A included 4096 unique combinations of variables derived from all possible combinations of the available: 8 measures of alcohol use, 8 measures of emotional problems, 16 confounder combinations, 2 types of missing data, and 2 time points. Multiverse B had 3584 specifications as early alcohol use was not included as an outcome in this set of analyses. Each specification (otherwise known as a universe or an individual regression model) is analyzed separately through linear (for continuous outcome specifications) or logistic (for binary outcome specifications) regression with standard errors adjusted for school clustering.

All continuous variables were standardized before analysis to enable comparisons across specifications. Standardized effects of 0.01, 0.2, 0.5, and 0.8 are often interpreted as very small, small, medium, and large effects respectively (Matthay et al., 2021; Sullivan & Feinn, 2012). Given the scaling of residuals changes across nested logistic models depending on the model fit, which rescales coefficients accordingly (resulting in non-collapsibility; [Schuster, Twisk, Ter Riet, Heymans, & Rijnhart, 2021]), the log-odds obtained from logistic models were y-standardized to ensure appropriate comparability across nested models (Williams & Jorgensen, 2023). This was accomplished through dividing the raw logistic regression coefficients by the estimated standard deviation of y* (i.e. the continuous latent dependent variable assumed to underly the dichotomous variable) (Huang, 2023).

First, the proportion of specifications where *p* < 0.05 are reported (Steegen et al., 2016). From the SCA framework, results are visualized through specification ‘curves’ that demonstrate the direction and (in)consistency in the magnitude of effects across specifications by plotting effects in order of magnitude (Simonsohn et al., 2020). From the VoE framework (Patel et al., 2015), volcano plots demonstrate the degree of (in)consistency in the direction of effects (provided in online Supplementary materials), and descriptive summary statistics are presented including the median beta, Range of Betas (RBs) depicting the range of standardized beta coefficients, the median *p* value (50th%), and the Range of -log10(*p* values) (RPs) presenting the *p* values between the 1st and 99th percentiles. Larger ranges in both RBs and RPs suggest greater variability across the universe. Further, the variances in effects were decomposed by parameter specification, by: (1) calculating intra-class correlation coefficients (ICCs) for each overarching specification, and (2) plotting median and interquartile range of betas for each specification using box and whisker plots. These approaches protect against selective reporting and *p* hacking by presenting all justifiable specifications available.

## Results

### Descriptive characteristics

The sample includes 49% female and 12% culturally and linguistically diverse adolescents. The prevalence of most alcohol specifications nearly doubled from T4 to T5. Emotional problems also showed a slight increase between T4 and T5. See Table 2 for details.

**Table 2.** Descriptive Alcohol Use and Emotional Problem Sample Characteristics

	Complete Case				Multiply Imputed (mean across imputations)	
	T4	%missing from total T4 sample	T5	%missing from total T5 sample	T4	T5
Alcohol Use						
Past 6-months any alcohol use	16.0%	29.2%	29.9%	36.4%	17.4%	38.2%
Past 6-months ~monthly alcohol use	7.3%		14.1%		5.3%	9.5%
Past 6-months frequency of alcohol use	0.3 (0.9)		0.5 (1.1)		0.3 (0.8)	0.6 (0.9)
Past 6-months binge drinking	7.0%	29.7%	14.4%	37.0%	7.0%	14.2%
Past 6-months ~monthly binge drinking	3.5%		6.7%		2.6%	4.4%
Past 6-months average monthly alcohol volume (grams/centiliters, log transformed)	0.4 (1.3)	29.2%	0.9 (1.7)	36.4%	0.5 (1.1)	0.9 (1.4)
Past 6-months alcohol problems	1.4 (4.9)	29.3%	1.9 (5.3)	36.5%	1.5 (4.2)	2.1 (4.3)
Early alcohol use (⩽14 years of age)	12.3%	15.0%	12.3%	14.9%	12.5%	12.6%
Emotional Problems						
Psychological distress symptoms (K6)	8.1 (6.2)	29.9%	8.4 (6.4)	36.8%	8.2 (5.5)	8.7 (5.5)
High psychological distress (K6 ⩾ 13)	22.5%		23.8%		19.6%	21.0%
Emotional symptoms (SDQ-E)	3.4 (2.8)	31.8%	3.9 (2.7)	39.0%	3.5 (2.5)	3.9 (2.3)
Abnormal emotional symptoms (SDQ-E ⩾ 7)	15.3%		18.2%		12.2%	14.0%
Depressive symptoms (PHQ-A)	6.6 (6.5)	30.4%	6.8 (6.5)	37.2%	6.7 (5.7)	7.1 (5.5)
Moderate to severe depressive symptoms (PHQ-A ⩾ 10)	27.5%		28.1%		25.2%	27.7%
Anxiety symptoms (PROMIS-A)	24.9 (12.7)	30.6%	25.2 (12.6)	37.3%	25.1 (11.2)	25.8 (10.8)
Moderate to severe anxiety symptoms (PROMIS-A ⩾ 34)	22.9%		24.2%		20.0%	21.3%

### Multiverse A: alcohol use predicting emotional problems

Overall, there was a considerable range of effects (RBs = 0.67; RPs = 29.38) with less than half of the models yielding significant alcohol effects (45.56% significant; 38.11% positive, 7.45% inverse) on emotional problems. Effects not only ranged in magnitude but also direction, with the full RBs from −0.41 to 0.69 (See Fig. 1a and Extended Data 4A).

**Figure 1.**
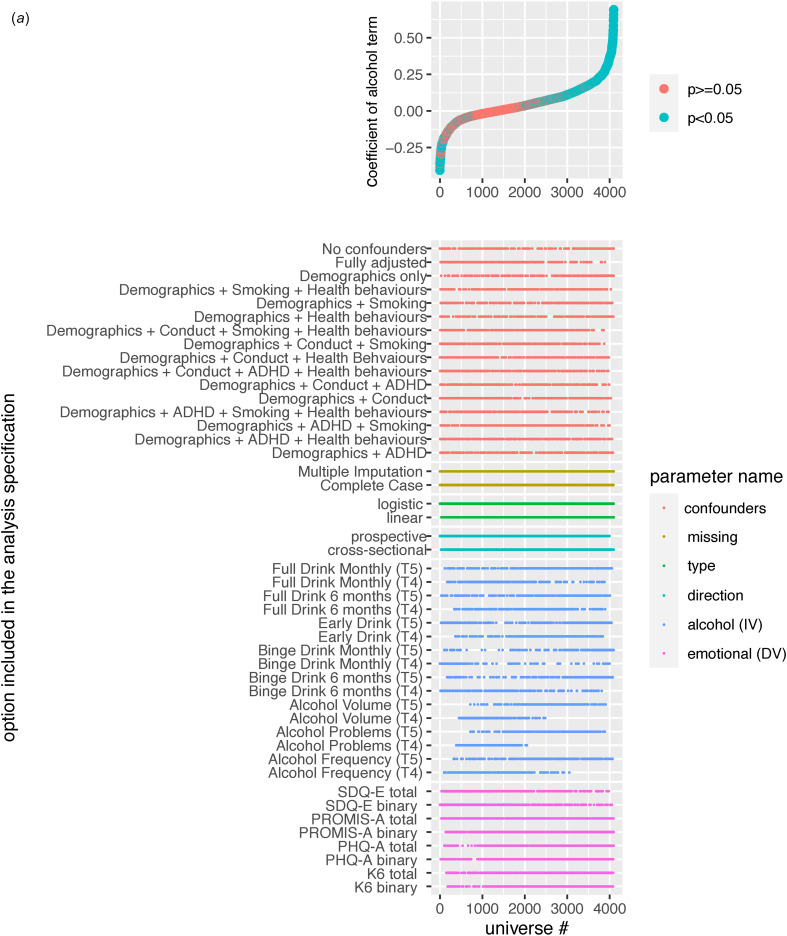
(a) Specification Curve Multiverse A. The specification curve plot in the top part of the figure plots standardized alcohol parameter estimates from each universe in multiverse A from smallest to largest. The blue dots represent parameter estimates that are statistically significant at a *p*<0.05. The red dots represent parameter estimates that are not statistically significant. Dots below “0” indicate models in which higher levels of the alcohol variable are associated with lower emotional problems. Dots above “0” indicate models in which higher alcohol is associated with higher emotional problems. The detail below the graph shows the universe where each specification listed on the left is included (i.e., the dot in the top figure lines up with dots in the detailed section below to indicate which specifications were in that particular model).

**Figure 1.**
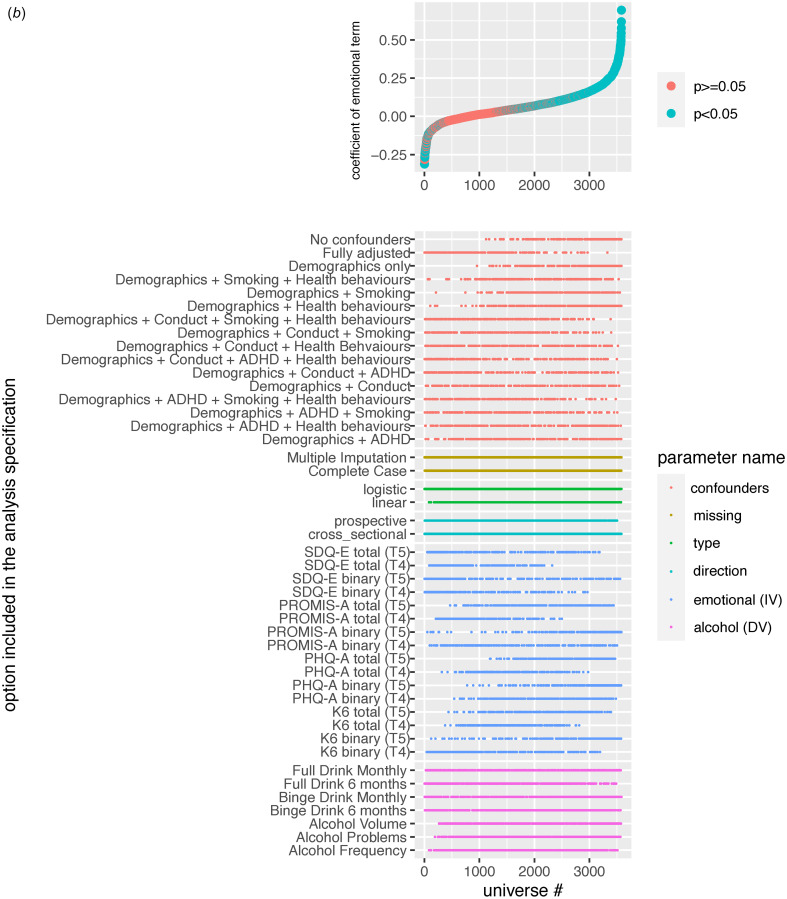
Specification Curve Multiverse B. The specification curve plot in the top part of the figure plots standardized emotional problems parameter estimates from each universe in multiverse B from smallest to largest. The blue dots represent parameter estimates that are statistically significant at a p<0.05. The red dots represent parameter estimates that are not statistically significant. Dots below “0” indicate models in which higher values on the emotional problems variable are associated with lower values on the alcohol variable. Dots above “0” indicate models in which higher emotional problems is associated with higher values on the alcohol variable. The detail below the graph shows the universe where each specification listed on the left is included (i.e., the dot in the top figure lines up with dots in the detailed section below to indicate which specifications were in that particular model).

Variability across these models was attributed to whether the model was specified cross-sectionally or prospectively (ICC = 0.30), the operationalization of emotional problems (ICC = 0.16) and alcohol use (ICC = 0.15), and the confounder adjustments (ICC = 0.11). Cross-sectional models consistently yielded larger significant positive effects while prospective models (after adjusting for prior levels of emotional problems)^†^^1^ were largely null with most significant effects found in the inverse direction (See Figs 2a, 3a, and Extended Data 4A). The alcohol specifications that had the smallest relative change in effects from cross-sectional to prospective specifications were: (1) any full drink in the past 6-months, and (2) early alcohol use. Operationalization of emotional problems that focused on depression (i.e. PHQ-A), or had more depression than anxiety items (i.e. K6), yielded consistently larger significant positive effects compared to those focused on anxiety (i.e. PROMIS-A), or with more anxiety than depression items (i.e. SDQ-E). The only outcome that yielded more significant inverse effects (23–25%) than positive effects (13–17%) was the SDQ-E.

**Figure 2.**
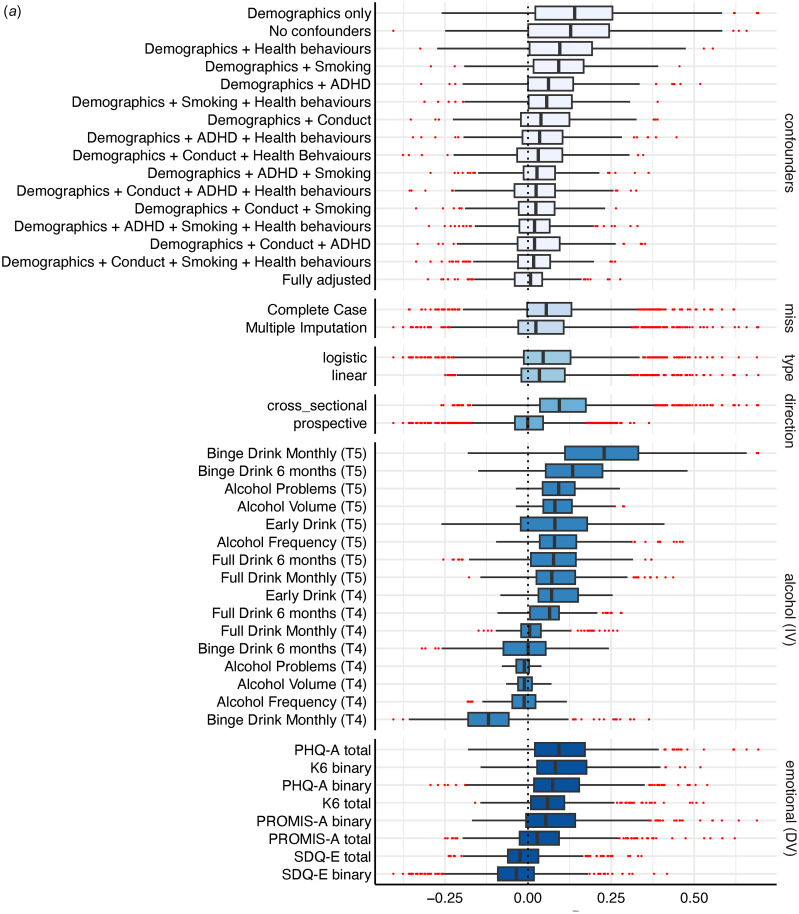
(a) Box Plot Multiverse A. The box plot shows the median eLect, interquartile range, and full range of all universes (or models) where the specification indicated on the left side of the figure was included. For example, Binge Drink Monthly (T5) has a black line at the median eLect of 0.23, reflecting the median eLect across all crosssectional specifications where Binge Drinking Monthly was included, The blue box around the median eLect is bound by the eLect at the 25_th_% (here, 0.11) and the 75_th_% (here, 0.33). The whiskers reflect the lower and upper data points within 1.5*the interquartile range. For Binge Drink Monthly (T5) this is -0.18 (as that is the lowest observed value) at the lower end and 0.66 at the upper end. The red dots reflect outliers beyond these edges.

**Figure 2.**
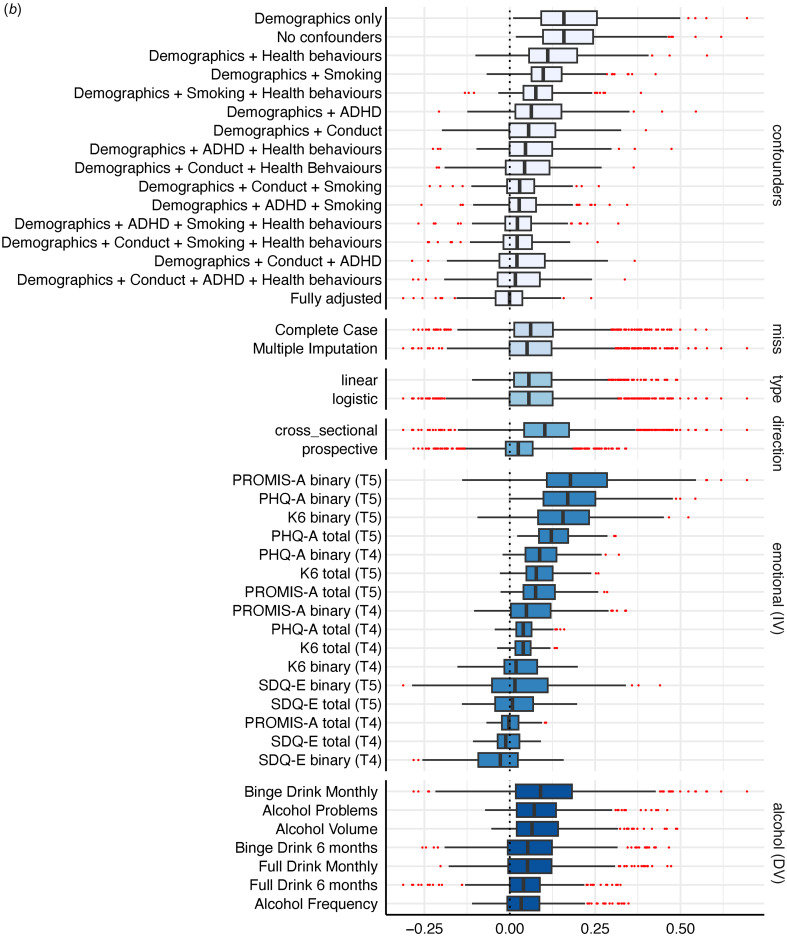
The box plot shows the median eLect, interquartile range, and full range of all universes (or models) where the specification indicated on the left side of the figure was included. For example, PHQ-A binary (T5) has a black line at the median eLect of 0.17, reflecting the median eLect across all crosssectional specifications where PHQ-A binary was included. The blue box around the median eLect is bound by the eLect at the 25_th_% (here, 0.10) and the 75_th_% (here, 0.25). The whiskers reflect the lower and upper data points within 1.5*the interquartile range. For PHQ-A binary (T5) this is 0.002 at the lower end (as that is the lowest observed value) and 0.48 at the upper end. The red dots reflect outliers beyond these edges.

**Figure 3.**
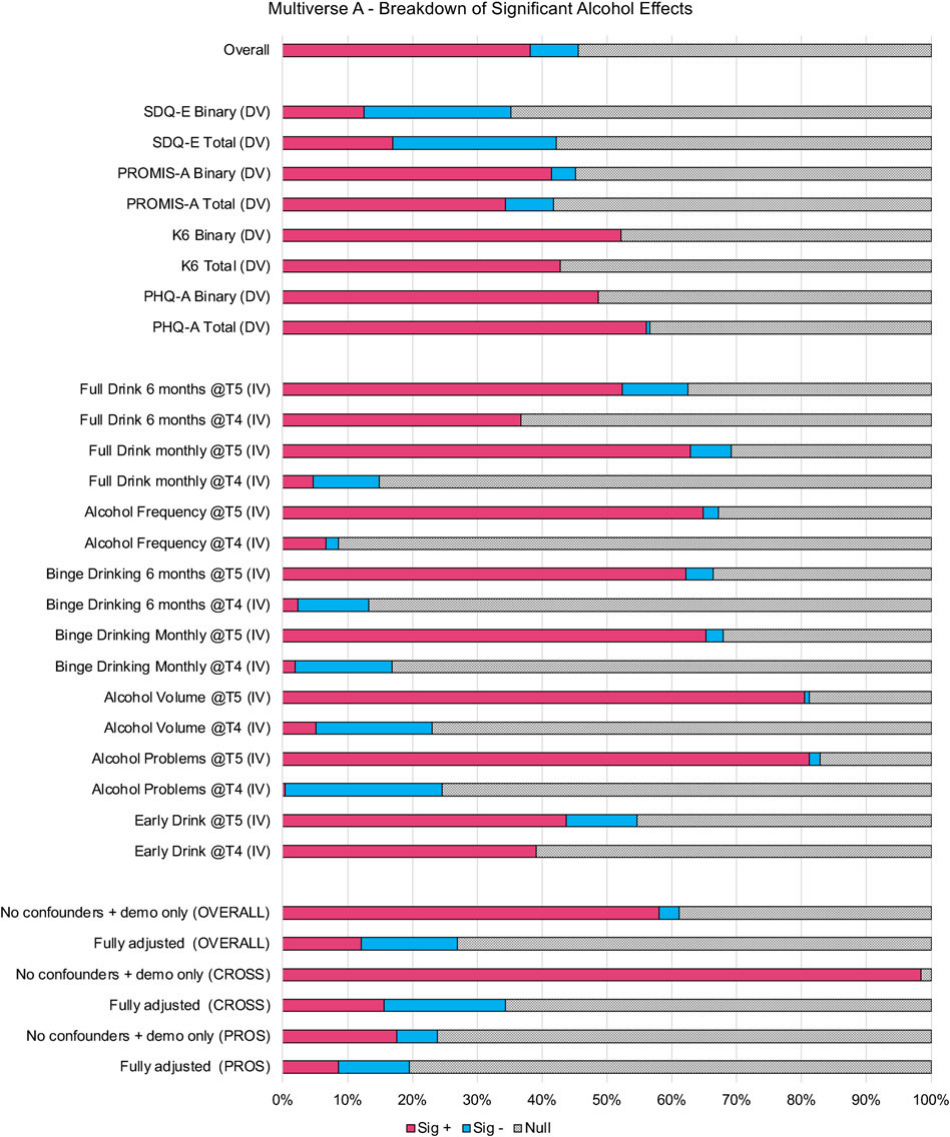
(a) *p* value Plot Multiverse A. This figure shows the proportion of statistically significant alcohol eLects (*p*<0.05) that are positively related to emotional problems (Sig +) and inversely related to emotional problems (Sig -) of all universes (or models) where the specification indicated on the left side of the figure was included.

**Figure 2.**
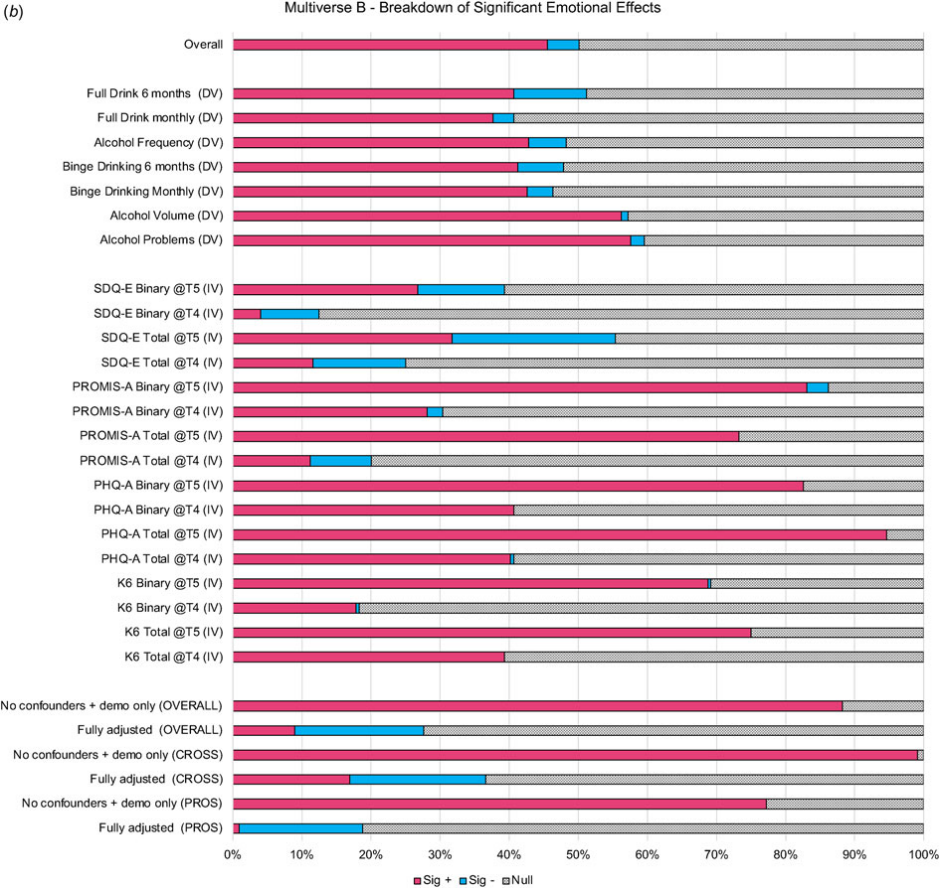
P-value Plot Multiverse B. This figure shows the proportion of statistically significant emotional problems eLects (*p*<0.05) that are positively related to alcohol variables (Sig +) and inversely related to alcohol variables (Sig -) of all universes (or models) where the specification indicated on the left side of the figure was included.

Further, when examining the median and IQR across confounder specifications (See Fig. 2a), adjusting for conduct symptoms had the largest impact on mitigating the magnitude of alcohol effects, followed by ADHD symptoms and smoking. When adjusting for either no confounders or only demographic confounders, 61% of effects across all specifications were significantly positively related (98% of cross-sectional, 24% of prospective; See Fig. 3a and Extended Data 4A). In fully adjusted models, only 27% were significantly related with just over half of these effects (14% overall) being inversely related; for cross-sectional 34% were significant in fully adjusted models with 16% positively and 19% inversely related, while in prospective models 20% were significant with 9% positively and 11% inversely related.

### Multiverse B: emotional problems predicting alcohol use

Overall, there was a considerable range of effects (RBs = 0.57; RPs = 20.65) with only half of the models yielding significant emotional problem effects (50.14% significant; 45.54% positive, 4.6% inverse) on alcohol use. Again, effects ranged in both magnitude and direction, with the full RBs going from −0.31 to 0.69 (See Fig. 1b and Extended Data 4B).

Variability across these models was attributed to whether the model was specified cross-sectionally or prospectively (ICC = 0.22), the operationalization of emotional problems (ICC = 0.25), and the confounder adjustments (ICC = 0.25). In this multiverse, the operationalization of alcohol use had little impact on the variability of effects across models (ICC = 0.04). Here, cross-sectional models consistently yielded larger significant positive effects while prospective models yielded attenuated effects (after adjusting for prior levels of alcohol use) that were largely null (See Figs 2b, 3b, and Extended Data 4B). Most depression-focused emotional problem specifications (i.e. total and binary PHQ-A, and total K6 specifications) still yielded ~40% significant positive effects in prospective models and <1% of any model with a depression-focused emotional specification had significant inverse effects. Effects related to anxiety-focused specifications were mixed and inconsistent. Similar to multiverse A, SDQ-E specifications yielded a non-negligible proportion of significant inverse effects in both cross-sectional and prospective models (8–24%). However, while cross-sectional effects related to PROMIS-A specifications were attenuated in prospective models with some significant inverse effects (3–9%), the pattern of associations with PROMIS-A as compared to SDQ-E scores were quite distinct.

Further, when examining the median and IQR across confounder specifications (See Fig. 2b), it appears as though adjusting for conduct symptoms had the largest impact on mitigating the magnitude of emotional problem effects, followed by ADHD symptoms. When adjusting for either no confounders or only demographic confounders, 88% of effects across all specifications were significant positively related (99% of cross-sectional, 77% of prospective; See Fig. 3b and Extended Data 4B). In fully adjusted models, only 28% were significantly related with a majority of these effects (19% overall) being inversely related; for cross-sectional 36% were significant in fully adjusted models with 17% positively and 19% inversely related, while in prospective models 18% were significant with nearly all inversely related.

## Discussion

This study explored nearly 8000 different ways of modelling the relationship between alcohol use and emotional problems, leveraging a recent sample of over 6000 adolescents. By using contemporary statistical frameworks to compare various specifications of these relationships within the same sample, we can draw conclusions about the co-occurrence of alcohol use and emotional problems among adolescents (regardless of measurement) and identify which measurement and analysis choices made by researchers impact findings. Echoing inconsistencies observed across different samples and studies in existing literature (Cochrane Canada, 2022; Dyer et al., 2019; Greenwood et al., 2021; Hussong et al., 2017; McCabe et al., 2023; Ning et al., 2020; Puddephatt et al., 2022; Watson et al., 2022), this multiverse analysis found notable inconsistencies within a single sample, depending on specifications. Methodologically, researcher decisions that appeared to have the biggest impact on findings included the operationalization of emotional problems, temporality of relationships, and choice of confounders. Researcher decisions that had minimal impact on findings were related to missing data strategies and whether outcomes were modelled continuously *v.* dichotomously. Inconsistencies in the magnitude, direction, and significance of effects between alcohol use and emotional problems appear closely tied to researcher decisions that are often regarded as relatively arbitrary.

The operationalization of emotional problems impacted the direction and magnitude of effects found, regardless of the direction of analysis, with depression-related measures more consistently positively related to alcohol use than anxiety-related measures. This suggests that there may be distinct associations between alcohol use and depression *v.* anxiety, indicating a broad ‘internalizing’ factor may inadequately capture these relationships during mid-adolescence. This is similar to previous studies that have found more consistent positive associations between adolescent alcohol and depression when compared to anxiety or general internalizing measures (Greenwood et al., 2021; Hussong et al., 2017; Ning et al., 2020). This may be because adolescent alcohol use typically occurs in a social context (Brooks-Russell, Simons-Morton, Haynie, Farhat, & Wang, 2014), which may be a barrier for use among adolescents with high levels of anxiety. The specific measures also seemed to operate differently, particularly the SDQ-E. However, whether emotional problems were operationalized using symptom scores or binary clinical cut-points did not play a large role in the magnitude and significance of effects.

The operationalization of alcohol use appeared to impact effects when exploring whether alcohol use predicted emotional problems, but not vice versa. Where emotional problems predicted alcohol use, there appeared to be general effects whereby the type, pattern, or measure of alcohol use did not seem to meaningfully impact the direction, magnitude, and significance of results. However, when alcohol use predicted emotional problems, associations across different operationalizations became more nuanced. For example, cross-sectionally, binge drinking had the strongest association with emotional problems, though these largely became null (or inverse) when prospectively explored. While it is possible that binge drinking may confer social and emotional benefits due to typical use in social settings (Brooks-Russell et al., 2014), most prospective models were null (83–87% null) and co-occurrence cross-sectionally was common. Prospectively, any past 6-month full standard drink consumption and early drinking seemed to remain the most consistently, positively related to later emotional problems (though still <50% significant). While previous literature has suggested differential associations depending on the operationalization of alcohol use (Dyer et al., 2019; Hussong et al., 2017; Puddephatt et al., 2022), this may be impacted by the stage of development (e.g. stronger prospective relationships earlier in adolescence) and direction of specified effects (Lees, Meredith, Kirkland, Bryant, & Squeglia, 2020; Spear, 2018).

The strongest sources of inconsistency across models related to the temporality, directionality, and included confounders of the specified model. Nearly universally, alcohol use and emotional problems cross-sectionally co-occurred (e.g. 98–99% of minimally adjusted cross-sectional models had significant, positive effects). Prospectively, alcohol use did not typically predict emotional problems 1 year later after accounting for baseline emotional problems (e.g. 76% null effects), and while prospective associations between emotional problems and alcohol use 1 year later were more consistent (e.g. 77% positive significant effects), these effects were strongly influenced by other researcher decisions (e.g. confounders, operationalization of outcomes). This aligns with existing literature suggesting weak or null prospective relationships between alcohol and emotional problems (Cochrane Canada, 2022; McCabe et al., 2023) with more evidence, though still nuanced, for emotional problems predicting alcohol use (Dyer et al., 2019; Hussong et al., 2017). What was evident was that the relationship between adolescent alcohol use and emotional problems was strongly influenced by choice of confounders, which may be due to true confounding or overcontrolling of higher-level constructs (e.g. 72–73% of fully adjusted models yielded null effects). For example, externalizing symptoms (e.g. conduct and ADHD symptoms) and other substance use (i.e. smoking) explained a large proportion (or all) of the shared variance between alcohol use and emotional problems.

Observational data presents a significant challenge due to the potential risk of residual confounding. The selection of appropriate confounders is thus crucial, though often underappreciated, researcher decision (Digitale, Martin, & Glymour, 2022; Herbert, 2020; Von Elm et al., 2007). There is both a risk for under- and over-controlling, with the ultimate goal to control for shared causal factors (e.g. factors that, when unaccounted for, suggest causal relationships that do not exist) while avoiding controlling for factors that lie on the causal pathway (e.g. mediators, or factors that explain causal relationships that do exist) (Herbert, 2020). While the selection of confounders for this multiverse adhered to established guidelines, in that they were selected *a priori* based on existing literature where they are commonly included and justified as confounders (Hernán, 2018; Larsson, 2022; Lederer et al., 2019), it is possible some specifications are at risk for over-controlling. For example, conduct symptoms, ADHD symptoms, and smoking appeared to explain a substantial amount of the relationship between alcohol use and emotional problems. While some researchers consider these critical confounders for alcohol-emotional relationships (Hussong et al., 2017), others view them as subdomains of overarching constructs (e.g. general externalizing, general substance use) suggesting controlling for these factors may inadvertently hide causal effects (Krueger et al., 2021; Vanyukov et al., 2012). To note, the aforementioned nuanced differences for anxiety and depression (*v.* general internalizing) provide evidence against higher-order constructs (Watson et al., 2022)–thus, further exploration into specific lower and higher-order constructs across different developmental stages is needed.

Two types of modeling specifications that often receive considerable attention by epidemiologists had minimal to no impact on effects in these data: (1) the approach to dealing with missing data, and (2) logistic *v.* linear models. First, the multiverse explored what is typically regarded as poor practice (i.e. complete cases analysis) compared to best practice (i.e. multiple imputation) (Enders, 2017). In our sample, for this question, how we treated missing data only explained <2% of the variability in the magnitude and direction of effects across specifications. As such, the effects did not seem to be biased depending on missing data practices and/or we did not have good enough auxiliary variables to predict missing data (e.g. it is possible data were missing not at random [MNAR]). There are other contemporary missing data approaches that may have been better suited to filling in the missing data in this sample, though these are often model-specific making them not feasible to apply to a multiverse of models (Enders, 2023). Second, once logistic effect estimates were y*standardized, the model function had largely null effect on the magnitude of effects across models. Notably, when y*standardization was not applied, logistic models seemingly yielded significantly larger effects than linear models. As such, while correct interpretation of beta coefficients from logistic models is critical (Williams & Jorgensen, 2023), dichotomization of variables of interest did not appear to lose meaningful explanatory power.

There are limitations to consider when interpreting this multiverse analysis. First, while the Health4Life dataset is a large, contemporary sample spanning three Australian states, it is not a representative sample. Second, data were collected in 2021 and 2022; though 2022 was not impacted by COVID19 lockdowns, there were a number of lockdowns in 2021 which may have impacted prospective associations. Third, these analyses give equal weighting to all specifications (Simonsohn et al., 2020) and there are several circumstances where the same question was operationalized in multiple different ways (e.g. full alcohol drink past 6 months, past month, frequency). Fourth, several significant findings may be a reflection of type 1 errors due to multiple testing; as these approaches look at overall distributions of effects and *p* values, rather than specific models, this is not a major concern. Fifth, this multiverse does not include *all* conceivable specifications of this relationship. For example, the Health4Life study did not include any diagnostic assessments of emotional disorders, measures of suicidality, or specific types of anxiety disorders. We also only explored one cut-off for each measure. Certain confounders were also not measured in Health4Life and thus could not be evaluated such as cannabis use, e-cigarette use, or family history. We also did not explore other types of covariates, such as moderators or mediators and only used one (cross-sectional) or two (prospective) time points – reflecting the most commonly evaluated models in the broader literature – precluding adjusting for confounders measured prior to the exposure and time-varying confounders (Cinelli, Forney, & Pearl, 2022; Clare, Dobbins, & Mattick, 2019). Notably, these multiverse models did not follow a formal causal inference framework, as the goals were to explore the impact of different specifications that are and have been commonly reported on in the literature. Further, outcomes were analyzed using linear and logistic models, though it is possible other approaches may have fit certain specifications better (e.g. Poisson, log-binomial).

The key findings of this multiverse are: (1) alcohol use and emotional problems commonly co-occur among adolescents; (2) emotional problems, particularly depression, may predict later alcohol use among adolescents, but there is limited evidence for the reverse; (3) conduct symptoms, ADHD symptoms, and smoking explain most of the associations between adolescent alcohol use and emotional problems; and (4) researcher decisions related to the operationalization of variables, inclusion of confounders, and choice in temporality and underlying causal hypotheses influence the magnitude, direction, and significance of relationships found between adolescent alcohol use and emotional problems. Whether these are causal relationships requires the application of formal causal modelling approaches. Based on the current findings, practitioners and policymakers should consider both: (1) the most consistent findings (i.e. co-occurrence was common and emotional problems often preceded alcohol use among adolescents) and (2) the degree of inconsistency in findings and possible reasons for inconsistency where it exists (i.e. the relationships may be explained by other substance use or behavioral problems). This enables cautious and accurate interpretations, while remaining open to further research to clarify our understanding. The contemporary analytical multiverse framework used in this paper needs to be applied to different samples of adolescents to uncover methodological and substantive reasons for the current inconsistencies in the literature.

## Supporting information

Halladay et al. supplementary material Halladay et al. supplementary material

## Footnotes

† The notes appear after the main text.

## Note

1 Post-hoc exploratory analysis showed that when prior levels of emotional problems were not adjusted for in prospective models, alcohol variables were largely positively related to emotional problems. After adjusting for prior levels of emotional problems, they largely reversed in direction of effects.

## Supplementary material

The supplementary material for this article can be found at https://doi.org/10.1017/S0033291724002502

## Code availability

The code for this analysis is available open access of open science framework at DOI 10.17605/OSF.IO/G2EQD.
