# Supplementary material for: Across the multiverse: exploring a diverse set of specifications related to cross-sectional and prospective associations between adolescent alcohol use and emotional problems
Source: Psychol Med. 2024 Dec 16;54(16):4585–99. doi: 10.1017/S0033291724002502 (PMC11769910; doi:10.1017/S0033291724002502)

**SUPPLEMENTARY MATERIALS: Across the multiverse: Exploring a diverse set of specifications related to cross-sectional and prospective associations between adolescent alcohol use and emotional problems**

Halladay, Visontay, Slade, Devine, Smout, Andrews, Champion, Teesson, Sunderland

**Figure 4.A** Volcano Plot Multiverse A

**Figure 4.B** Volcano Plot Multiverse B

**Extended Data**

**Figure 4.A** Volcano Plots for Multiverse A


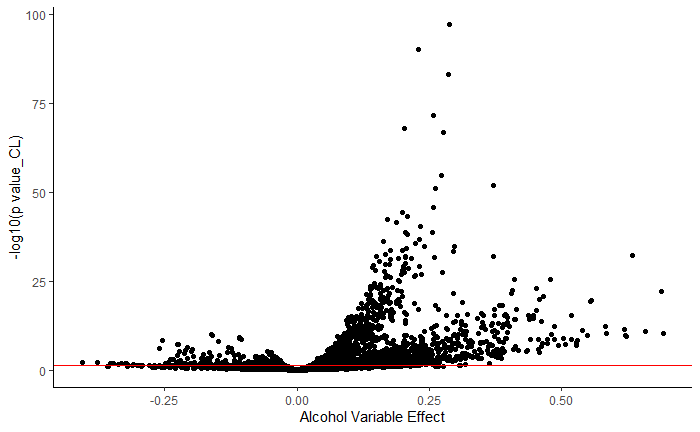


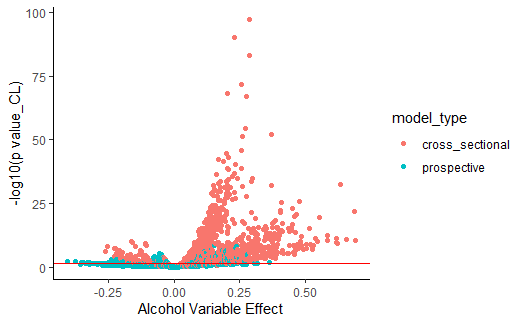


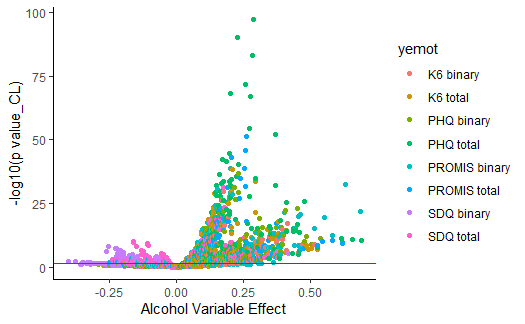


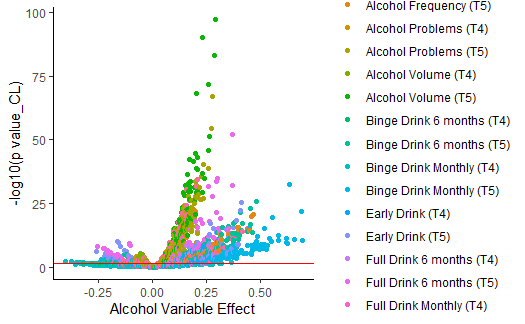


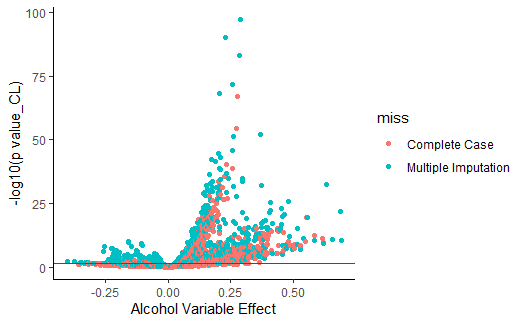


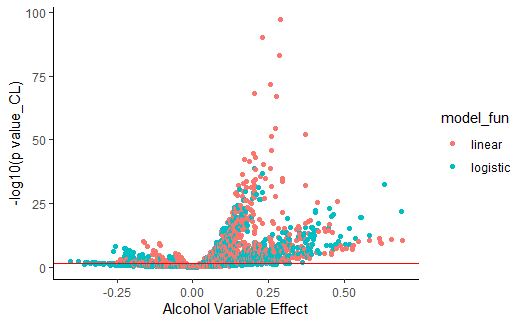


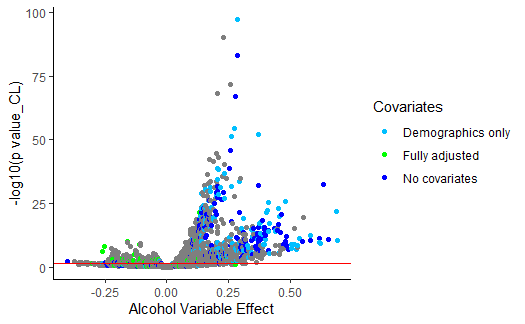


*note, the light grey dots are the models with other sets of confounder adjustments


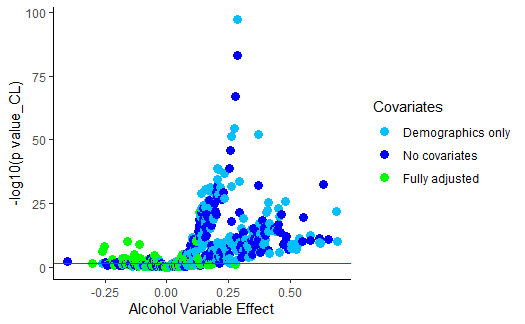


**Figure 4.B** Volcano Plot Multiverse B


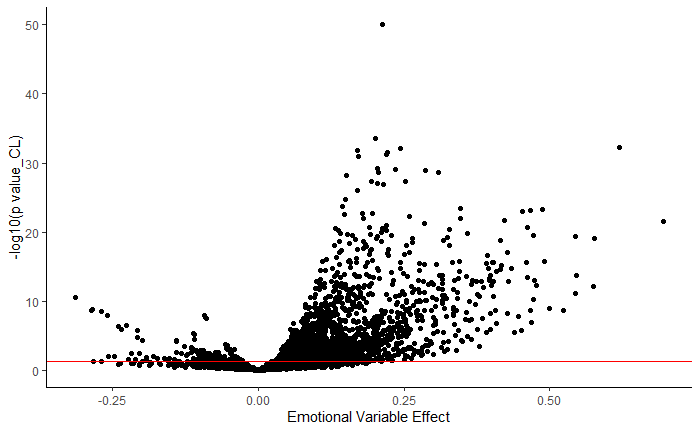


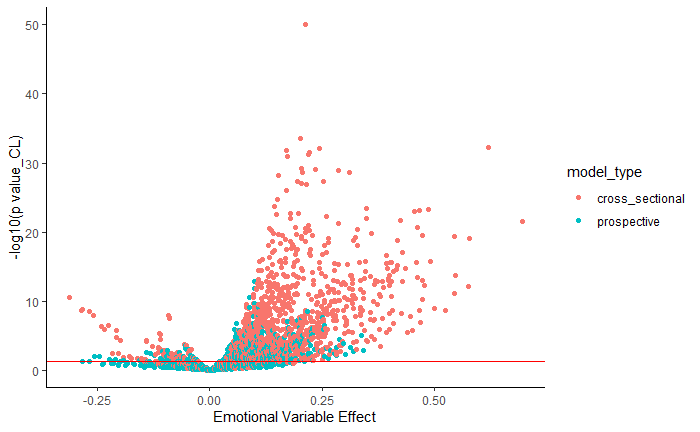


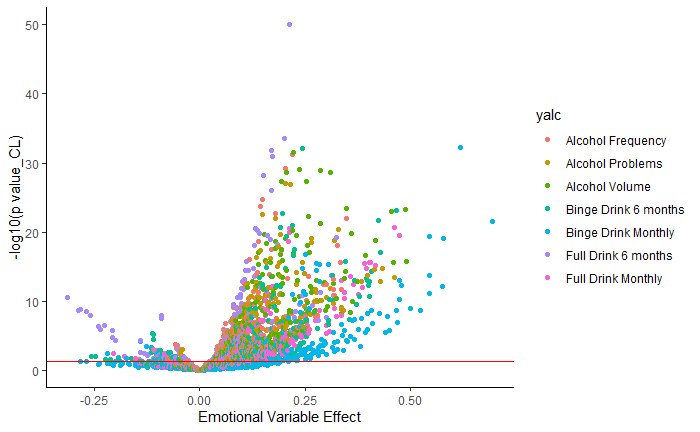


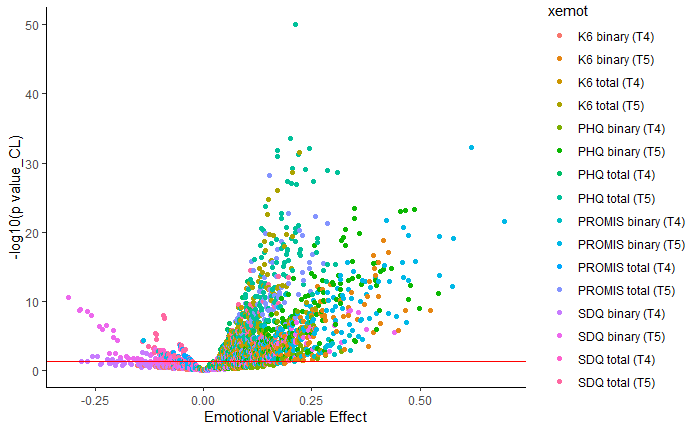


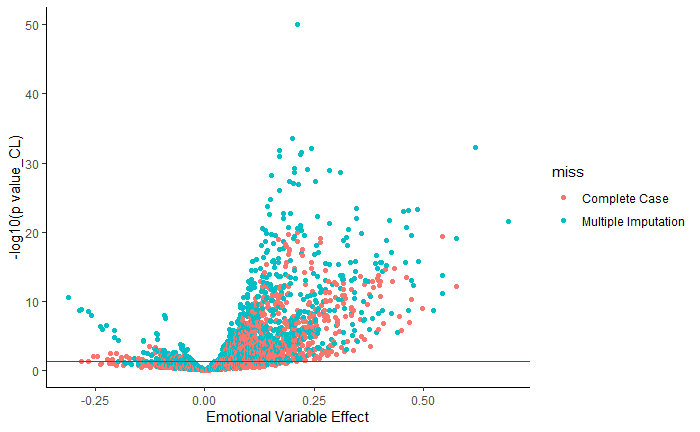


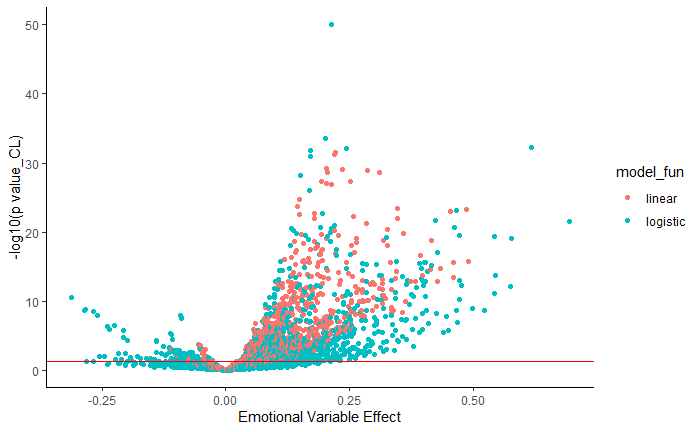


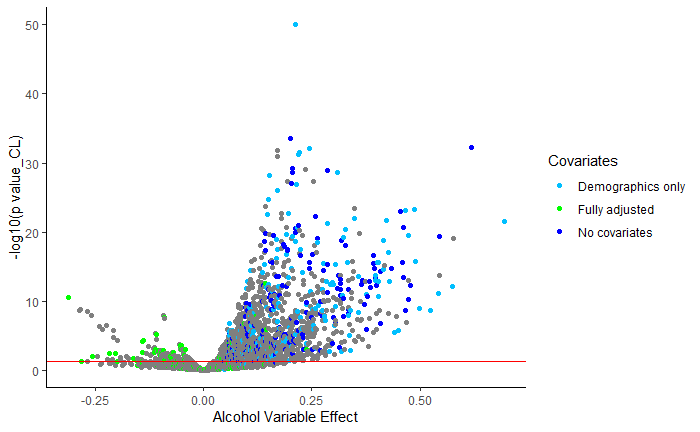


*note, the light grey dots are the models with other sets of confounder adjustments


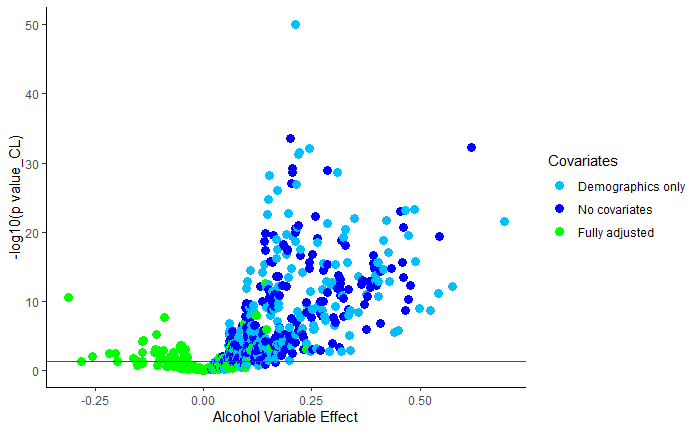

Supplement: Halladay et al. supplementary material [file S0033291724002502sup001.docx]
